# Decisive reversal of lethal coronavirus disease 2019 in senescent hamster by synchronic antiviral and immunoregulatory intervention

**DOI:** 10.1002/mco2.642

**Published:** 2024-07-19

**Authors:** Xuan Liu, Ming Zhou, Mujing Fang, Ying Xie, Peiwen Chen, Rirong Chen, Kun Wu, Jianghui Ye, Che Liu, Huachen Zhu, Tong Cheng, Lunzhi Yuan, Hui Zhao, Yi Guan, Ningshao Xia

**Affiliations:** ^1^ Clinical Center for Bio‐Therapy Zhongshan Hospital Fudan University (Xiamen Branch) Xiamen Fujian China; ^2^ State Key Laboratory of Vaccines for Infectious Diseases, National Institute of Diagnostics and Vaccine Development in Infectious Diseases, NMPA Key Laboratory for Research and Evaluation of Infectious Disease Diagnostic Technology, School of Life Sciences & School of Public Health Xiamen University Xiamen Fujian China; ^3^ National Institute for Food and Drug Control Beijing China; ^4^ Institute of Medical Biology Chinese Academy of Medical Science and Peking Union Medical College Kunming China; ^5^ State Key Laboratory of Emerging Infectious Diseases, School of Public Health, Li Ka Shing Faculty of Medicine The University of Hong Kong Hong Kong SAR China; ^6^ Guangdong‐Hong Kong Joint Laboratory of Emerging Infectious Diseases/Joint Laboratory for International Collaboration in Virology and Emerging Infectious Diseases, Joint Institute of Virology (STU/HKU) Shantou University Shantou Guangdong China

**Keywords:** antiviral therapy, bio‐mimic decoy, immunoregulation, SARS‐CoV‐2, senesce associated disease deterioration

## Abstract

The poor prognosis observed in elderly individuals infected with severe acute respiratory syndrome coronavirus 2 (SARS‐CoV‐2) remains a serious clinical burden and the underlying mechanism is unclear, which necessities detailed investigation of disease characteristics and research for efficient countermeasures. To simulate lethal coronavirus disease 2019 (COVID‐19) in senescent human patients, 80‐week‐old male hamsters are intranasally inoculated with different doses of SARS‐CoV‐2 Omicron BA.5 variant. Exposure to a low dose of the Omicron BA.5 variant results in early activation of the innate immune response, followed by rapid viral clearance and minimal lung damage. However, a high dose of BA.5 results in impaired interferon signaling, cytokine storm, uncontrolled viral replication, and severe lung injury. To decrease viral load and reverse the deterioration of COVID‐19, a new bio‐mimic decoy called CoVR‐MV is used as a preventive or therapeutic agent. Administration of CoVR‐MV as a preventive or therapeutic intervention in the early stages of infection can effectively suppress viral load, regulate the immune response, and rescue animals from death and critical illness. These findings underscore the risk associated with SARS‐CoV‐2 Omicron BA.5 exposure in senescent hamsters and highlight the importance of early intervention to prevent disease progression.

## Graphical abstract

This study reveals the dose‐dependent disease outcomes of SARS‐CoV‐2 Omicron BA.5 exposure in senescent hamster. High‐dose BA.5 exposure results in impaired IFN I signaling, cytokine storm, uncontrolled viral replication, severe lung injury, and death. Early intervention of bio‐mimic decoy CoVR‐MV rescues critical COVID‐19 by viral load inhibition and simultaneous reserving the imbalanced innate immune response from inflammatory to antiviral.

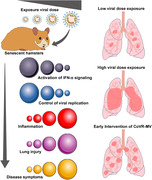

## 1 INTRODUCTION

The omicron variant lineage has emerged as the predominant strain worldwide during the ongoing pandemic caused by severe acute respiratory syndrome coronavirus 2 (SARS‐CoV‐2). SARS‐CoV‐2 infection can result in a range of manifestations, from asymptomatic cases to varying degrees of illness in coronavirus disease 2019 (COVID‐19) patients, including symptoms such as cough, weakness, pneumonia, acute respiratory distress syndrome (ARDS), and even death.^1^, ^2^ Compared with the prototype and earlier variants (Alpha, Beta, Gamma, and Delta), the initial Omicron BA.1/BA.2 variants had significantly lower intrinsic virulence.^3^, ^4^, ^5^, ^6^ However, later variants such as Omicron BA.4/BA.5, BQ, and XBB showed a significant increase in immune escape and intrinsic virulence.^7^, ^8^, ^9^, ^10^, ^11^ In addition, the ongoing mutations in SARS‐CoV‐2 raise concerns about the risk of breakthrough infection and disease exacerbation in immunocompromised populations.^12^, ^13^, ^14^

The lung is the primary organ targeted by SARS‐CoV‐2 infection and plays a significant role in its pathophysiology. The physiological state and immune microenvironment of lung tissue have a profound impact on the host response to SARS‐CoV‐2 infection and the resulting disease outcomes.^15^, ^16^ As individuals age, the presence of immunosenescence and inflammaging impairs the physiological state of lung tissue, leading to attenuated antiviral innate immune responses and increased production of proinflammatory cytokines.^17^ The aged individuals and animals have been shown to have more severe cases of COVID‐19 compared with adults, primarily due to the combined effects of underlying health conditions, declining physiological functions, and abnormal immune responses.^18^, ^19^, ^20^ For example, immunosenescence has been strongly associated with inadequate vaccine protection, severe pneumonia, and lung failure following SARS‐CoV‐2 infection.^21^, ^22^, ^23^ However, many aspects of the critical progression of COVID‐19 in the elderly population remain unclear, severely limiting the development of effective clinical therapies and the implementation of efficient countermeasures.

Therefore, it is essential to simulate disease characteristics in animal models that exhibit senescence, to delineate the process of disease deterioration, and to investigate the underlying immunopathological mechanisms. These efforts are critical to advancing our fundamental understanding of the infection courses and risk factors of different SARS‐CoV‐2 variants, host immune responses and the progression of COVID‐19 in elderly patients. On the other hand, the senescent animal model of SARS‐CoV‐2 infection provides an ideal platform for the development and evaluation of efficient countermeasures against critical COVID‐19.

To achieve these goals, we used the Syrian hamster as a highly sensitive animal model to study the pathology of SARS‐CoV‐2 infection. By administering different doses of SARS‐CoV‐2 Omicron BA.5 via intranasal inoculation to 80‐week‐old male hamsters, we were able to determine that the severity of the disease is determined by the level of viral exposure. In particular, exposure to a high dose of virus typically results in robust viral replication, inappropriate activation of the interferon (IFN) signaling pathway, and the onset of a cytokine storm during the early stages of COVID‐19. This ultimately leads to severe pneumonia, ARDS‐like symptoms, and death. Conversely, senescent hamsters exposed to a low dose of virus exhibit rapid virus clearance and mild symptoms within 7 days. An integrated analysis of physiological, pathological, virological, and immunological features has shown that controlling the viral load and balancing the immune response during the early stages of COVID‐19 are crucial to prevent disease deterioration. Therefore, we conducted prophylactic and therapeutic interventions using a newly developed bio‐mimic decoy called CoVR‐MV,^24^ which exhibits potent direct neutralization efficacy and immunoregulatory functions. Our data conclusively demonstrate that early intervention with CoVR‐MV is sufficient to rescue senescent hamsters from death and critical COVID‐19.

## 2 RESULTS

### 2.1 A high viral dose of SARS‐CoV‐2 Omicron BA.5 leads to death and severe cases of COVID‐19 in senescent hamsters

**FIGURE 1:**
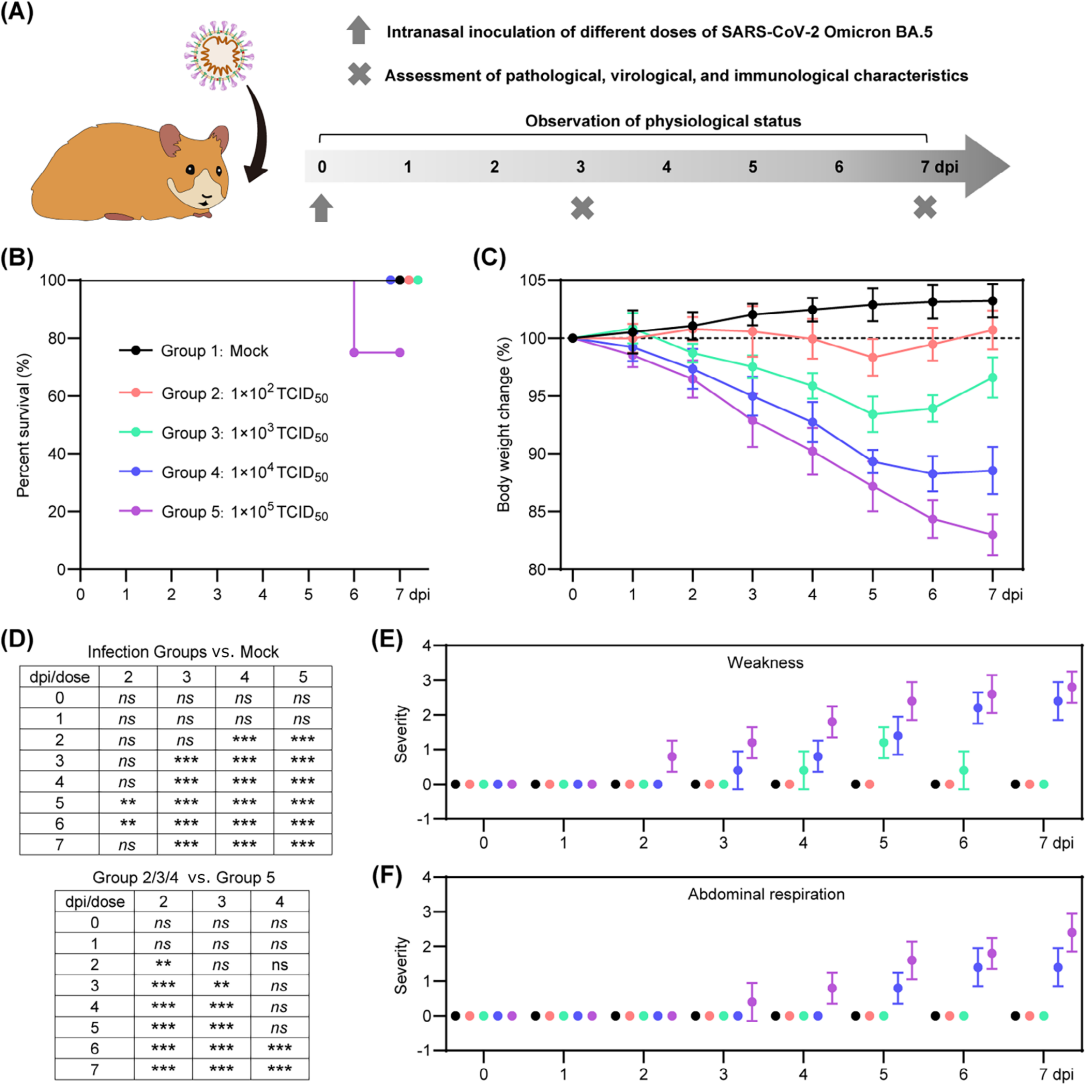
Physiological characteristics of the senescent hamsters after intranasal exposure to different doses of SARS‐CoV‐2 Omicron BA.5. (A) The schematic diagram shows the process of virus inoculation, symptom observation, and sampling from 0 to 7 dpi. (B) Survival rate is shown for mock senescent hamsters (group 1) and those inoculated with 1 × 10^2^ TCID_50_ (group 2), 1 × 10^3^ TCID_50_ (group 3), 1 × 10^4^ TCID_50_ (group 4), and 1 × 10^5^ TCID_50_ (group 5), respectively (*n* = 8). (C) Changes in body weight are shown from 0 to 7 dpi (*n* = 5). (D) Statistical analysis of body weight data for each group. Significance was calculated by two‐way ANOVA. Two‐sided *p* values < 0.01 were considered significant: *p < 0.01, **p < 0.001, ***p < 0.0001, ns indicates no significance. COVID‐19 symptom records include (E) weakness and (F) abdominal breathing (*n* = 5).

To investigate the effect of different viral exposure doses on the disease outcome of SARS‐CoV‐2 Omicron BA.5 infection, we performed intranasal virus inoculation in 80‐week‐old male hamsters. The hamsters were divided into five groups: group 1 served as a control group and did not receive any infection, whereas groups 2, 3, 4, and 5 were inoculated with viral doses of 1 × 10^2^ TCID_50_, 1 × 10^3^ TCID_50_, 1 × 10^4^ TCID_50_, and 1 × 10^5^ TCID_50_, respectively (Figure 1A). Throughout the animal experiment, which was recorded from 0 to 7 days postinfection (dpi), we monitored the survival rate, body weight loss, and physical condition of these senescent hamsters. In addition, a separate set of hamsters was sacrificed at 3 and 7 dpi to evaluate the pathological, virological, and immunological characteristics during the early and late stages of COVID‐19 progression.

Notably, at 7 dpi (Figure 1B), two of eight hamsters in group 5 succumbed to infection, whereas all hamsters in the other groups survived. Hamsters in groups 2 and 3 experienced moderate body weight loss from 0 to 5 dpi, followed by rapid recovery from 5 to 7 dpi (Figure 1C). In contrast, hamsters in groups 4 and 5 showed significant body weight loss throughout the period from 0 to 7 dpi (Figure 1C). Hamsters in groups 1 and 2 showed an increase in body weight of 3.2 ± 1.4 and 0.7 ± 1.6%, respectively, at 7 dpi (Figure 1C). On the other hand, hamsters in groups 3, 4, and 5 showed a body weight loss of 3.4 ± 1.7, 11.5 ± 2.1, and 17.0 ± 1.8%, respectively, at 7 dpi (Figure 1C). Overall, the hamsters exposed to a low viral dose experienced less body weight loss compared with those exposed to a high viral dose (Figure 1D). Furthermore, typical symptoms of COVID‐19 such as weakness (Figure 1E) and abdominal respiration (Figure 1F) were observed in hamsters from groups 4 and 5 between 3 and 7 dpi.

### 2.2 The severity of lung injury and the efficiency of viral clearance in senescent hamsters are dependent on the exposure viral dose

**FIGURE 2:**
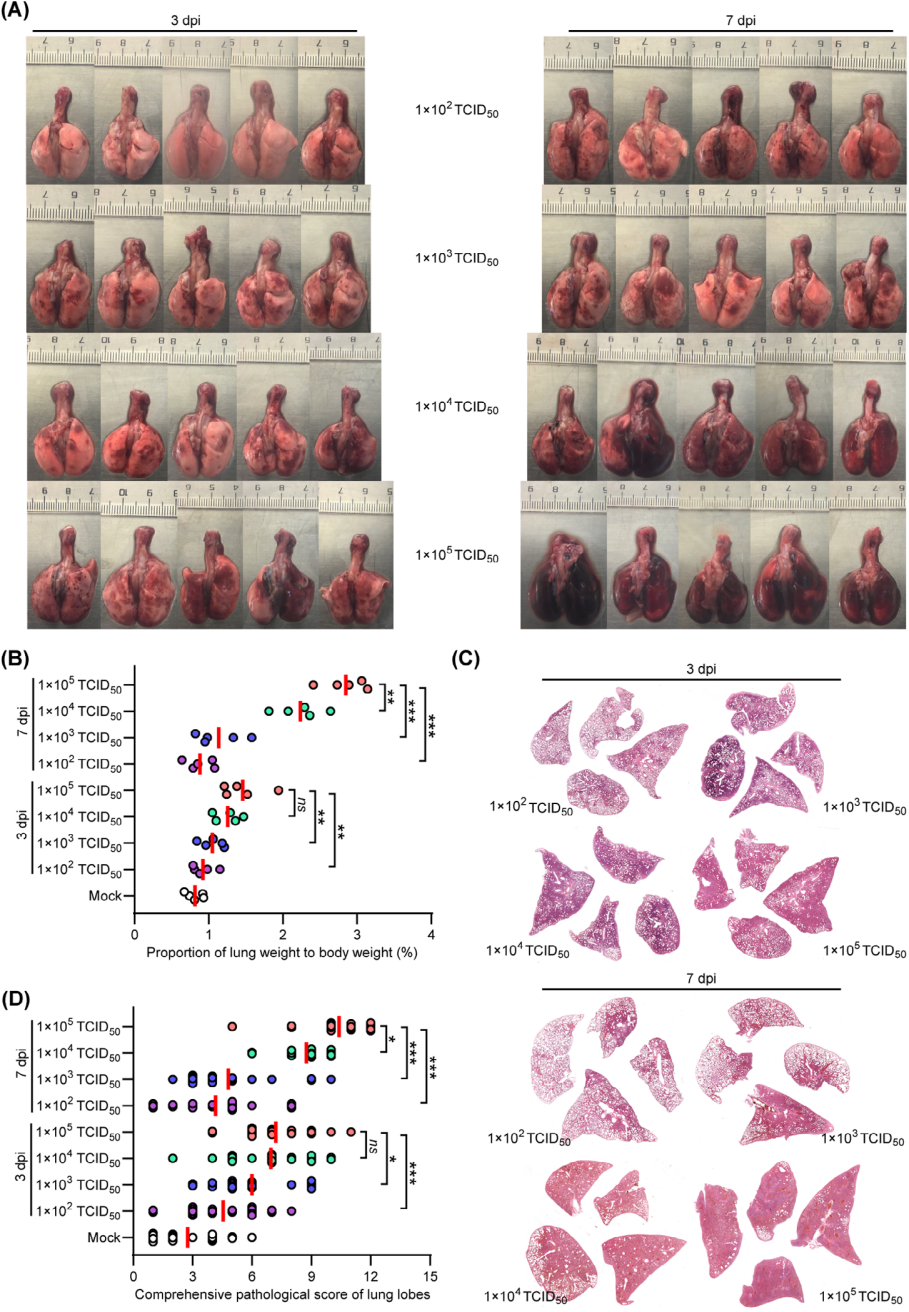
Pathological changes in the lung tissues of senescent hamsters following intranasal exposure of various doses of SARS‐CoV‐2 Omicron BA.5. (A) Gross images of lung tissues collected at 3 and 7 dpi (*n* = 5). (B) Lung weight to body weight ratio at 3 and 7 dpi (*n* = 5). (C) Representative H&E staining lung lobe sections collected at 3 and 7 dpi. H&E staining for the remaining hamsters is shown in Figure S1. (D) Comprehensive pathological scores were assigned to lung sections based on the severity and percentage of injured areas in each lung lobe. The mock hamsters without SARS‐CoV‐2 infection were euthanized at 7 dpi. Approximately 20 lung lobes from five individual hamsters were collected and scored for each group (Table S1). (B and D) Significance was determined by one‐way ANOVA. Two‐sided *p* values < 0.01 were considered significant: *p < 0.01, **p < 0.001, ***p < 0.0001, ns indicates no significance.

We then examined the pathological changes in lung tissues obtained from BA.5‐infected hamsters euthanized at 3 and 7 dpi. Gross images of lung tissues from groups 2 and 3 showed minimal and scattered lesion areas at both time points, which resembles the lung tissues of the mock hamsters that were euthanized at 7 dpi (Figures 2A and S1). In contrast, hamsters in groups 4 and 5 showed mild to severe lung injury at 3 dpi, which progressed to critical lung injury at 7 dpi (Figure 2A). Compared with the mock hamsters, there was a progressive increase in the ratio of lung weight to body weight from groups 2 to 5 in varied degrees (Figure 2B). Hematoxylin and eosin (H&E) staining of lung tissues revealed minimal to mild lung injury in certain lung lobes of the mock hamsters at 7 dpi, and those in groups 2 and 3 at 3 and 7 dpi (Figures 2C, S2, and S3). However, diffuse and critical lung injury was observed in over 90% of lung lobes in groups 4 and 5 at 3 and 7 dpi (Figures 2C, S2, and S3). In addition, the severity of lung pathology was assessed using comprehensive pathological scoring, which considered factors such as alveolar septal thickening and consolidation, hemorrhage, exudation, pulmonary edema, mucus accumulation, and inflammatory cell recruitment and infiltration in all lung lobes in each group (Figure 2D and Table S1). The average comprehensive pathological scores at 7 dpi were 2.8 ± 1.6, 4.2 ± 2.3, 4.8 ± 2.4, 8.8 ± 1.2, and 10.4 ± 1.8 for hamsters in groups 1–5, respectively (Figure 2D). These results indicate the likelihood of severe pneumonia in hamsters after high‐dose exposure to SARS‐CoV‐2 Omicron BA.5.

**FIGURE 3:**
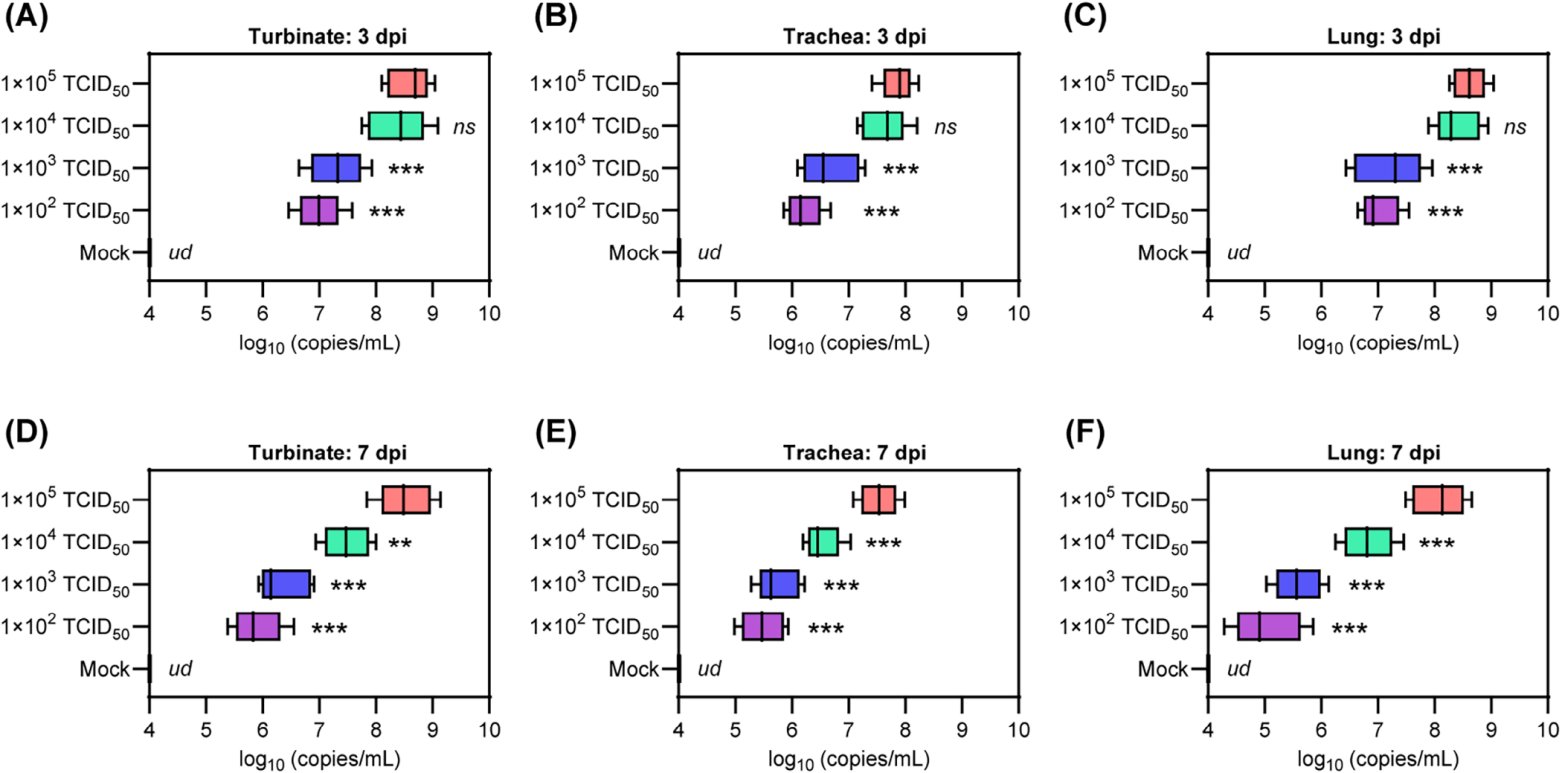
Viral RNA load in respiratory tract organs. Viral RNA levels in (A and D) nasal turbinate, (B and E) trachea, and (C and F) lung tissues that collected at 3 and 7 dpi were measured by RT‐qPCR, respectively (*n* = 5). Primers targeting the SARS‐CoV‐2 ORF1ab gene were used for amplification. Significance was determined by one‐way ANOVA. Two‐sided *p*‐values < 0.01 were considered significant: *p < 0.01, **p < 0.001, ***p < 0.0001, ns indicates no significance, and ud indicates undetectable.

Subsequently, we investigated the effect of viral exposure dose on viral replication in respiratory organs. To do this, we analyzed viral replication in specific organs, including the turbinate, trachea, and lung. This was done by performing real time quantitative polymerase chain reaction (RT‐qPCR) to amplify the SARS‐CoV‐2 ORF1ab gene, which allowed us to measure viral RNA levels in homogenized tissues collected at 3 and 7 dpi. At 3 dpi, the hamsters in groups 2–5 showed SARS‐CoV‐2 RNA levels of 7.0 ± 0.4, 7.3 ± 0.5, 8.4 ± 0.5, and 8.6 ± 0.4 in nasal turbinate (Figure 3A); 6.2 ± 0.3, 6.7 ± 0.5, 7.6 ± 0.4, and 7.9 ± 0.5 in trachea (Figure 3B); 7.0 ± 0.3, 7.2 ± 0.5, 8.4 ± 0.4, and 8.6 ± 0.3 log_10_ (copies/mL) in lungs, respectively (Figure 3C). At 7 dpi, the hamsters in groups 2–5 showed SARS‐CoV‐2 RNA levels of 5.9 ± 0.4, 6.4 ± 0.5, 7.5 ± 0.4, and 8.5 ± 0.5 in nasal turbinate (Figure 3D); 5.5 ± 0.4, 5.8 ± 0.4, 6.5 ± 0.3, and 7.5 ± 0.3 in trachea (Figure 3E); 5.0 ± 0.6, 5.6 ± 0.4, 6.8 ± 0.5, and 8.1 ± 0.5 log_10_ (copies/mL) in lungs, respectively (Figure 3F). Of note, the hamsters in groups 2–5 showed decrease of viral RNA levels of 84.3, 87.7, 89.3, and 98.8% in nasal turbinate; 88.7.5, 86.6.0, 85.5, and 94.9% in trachea; 71.4, 77.7, 80.9, and 94.2% in lungs from 3 to 7 dpi, respectively. Interestingly, hamsters in group 4 had similar viral RNA levels to those in group 5 at 3 dpi, but a significant decrease was observed at 7 dpi. These results suggest that the efficiency of viral clearance in the respiratory organs of senescent hamsters is influenced by the dose of viral exposure.

### 2.3 The senescent hamsters exhibit an imbalanced immune response after high viral dose exposure

**FIGURE 4:**
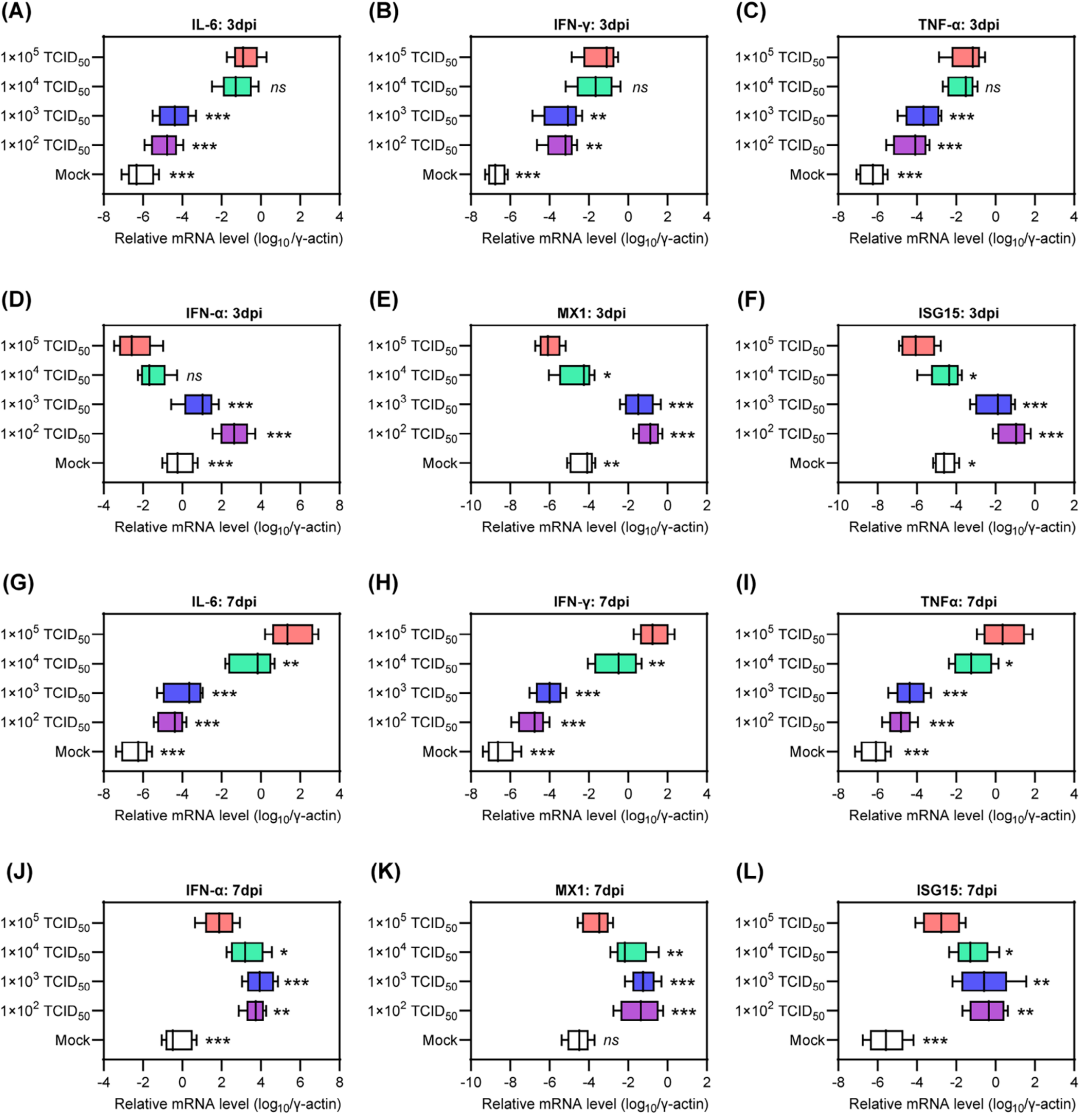
Changes of inflammation and innate immune response associated genes in lung tissues. The mRNA levels of proinflammatory cytokines including (A) IL‐6, (B) IFN‐γ, and (C) TNF‐α in the lung tissues that collected at 3 dpi, and (G–I) those collected at 7 dpi were measured by RT‐qPCR to determine the fold changes (*n* = 5). The mRNA levels of (D) IFN‐α and representative ISGs including (E) MX1 and (F) ISG15 in the lung tissues collected at 3 dpi and (J–L) those collected at 7 dpi were also measured by RT‐qPCR to determine the fold changes (*n* = 5). These mRNA levels were normalized to the housekeeping gene γ‐actin. Significance was determined by one‐way ANOVA. Two‐sided *p* values < 0.01 were considered significant: *p < 0.01, **p < 0.001, ***p < 0.0001, and ns indicates no significance.

Excessive inflammatory responses and uncontrolled viral replication are two distinct manifestations of critical COVID‐19 that are closely linked to an imbalanced host immune response.^25^, ^26^, ^27^, ^28^ Therefore, we evaluated the mRNA levels of proinflammatory cytokines, including interleukin‐6 (IL‐6), interferon gamma (IFN‐γ), and tumor necrosis factor alpha (TNF‐α), as well as genes associated with innate antiviral response, such as IFN alpha (IFN‐α), myxovirus resistance 1 (MX1), and IFN‐stimulated gene 15 (ISG15), in lung tissues collected at 3 and 7 dpi. Interestingly, at 3 dpi, hamsters in groups 2 and 3 had lower mRNA levels of IL‐6, IFN‐γ, and TNF‐α (Figure 4A–C), but higher mRNA levels of IFN‐α, MX1, and ISG15 compared with those in groups 4 and 5 (Figure 4D–F). These results indicate the presence of a priming inflammatory microenvironment and an impaired innate antiviral response in the lung tissue of senescent hamsters exposed to a high viral dose during the early stage of COVID‐19. Similarly, at 7 dpi, hamsters in group 5 exhibited excessive activation of the inflammatory response but insufficient innate antiviral response compared with those in groups 2 and 3 (Figure 4G–L). In contrast, hamsters in group 4 showed a significant decrease in IL‐6, IFN‐γ, and TNF‐α (Figure 4G–I) and an increase in IFN‐α, MX1, and ISG15 (Figure 4J–L) at 7 dpi, indicating a mitigation of critical COVID‐19. In general, senescent hamsters exposed to a low viral dose maintain upregulation of innate antiviral‐associated genes from 3 to 7 dpi, whereas these genes were suppressed by exposure to a lower viral load. Importantly, increasing viral dose exposure was associated with upregulation of proinflammatory cytokine genes.

### 2.4 The disease outcome of SARS‐CoV‐2 Omicron BA.5 infection in senescent hamsters is dependent on exposure viral dose

**FIGURE 5:**
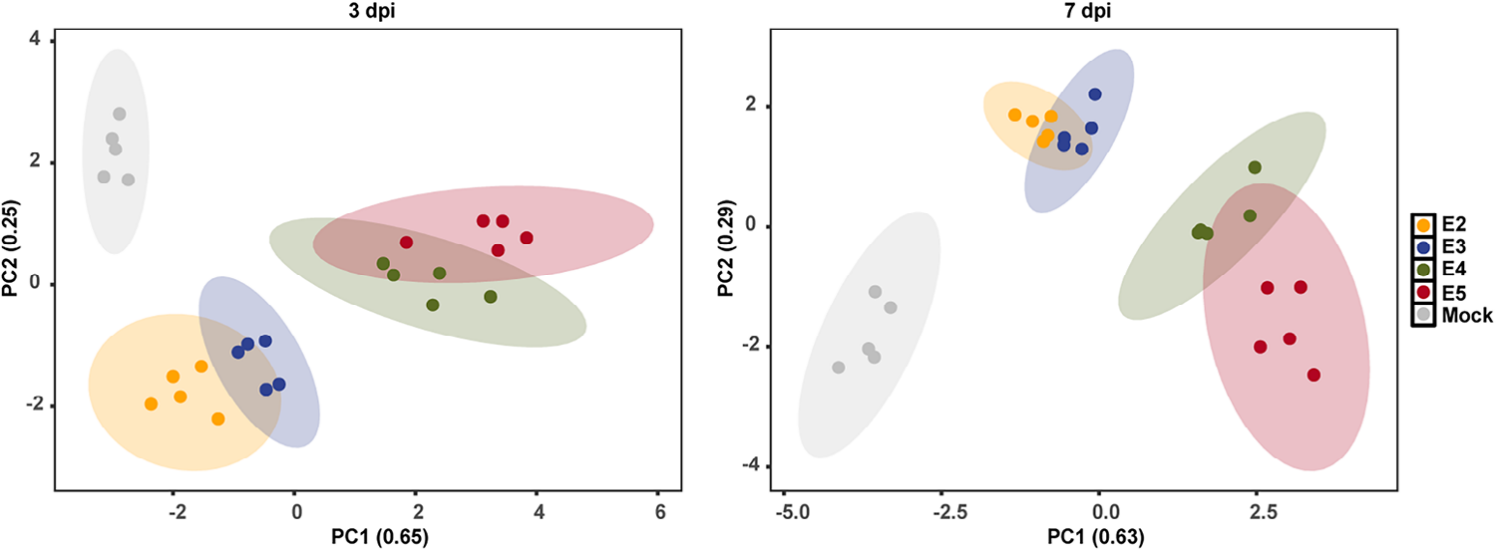
Comprehensive analysis of the relationship between viral exposure dose and disease outcome in senescent hamsters. The PCA plot indicates global differences among the mock and hamsters exposed to different doses of SARS‐CoV‐2 Omicron BA.5 variant (*n* = 5). The normalized values of body weight loss, viral RNA levels in lung tissues, pathological score of lung lobes, fold changes of proinflammatory cytokines, and innate antiviral response associated genes in lung tissues at 3 dpi (left panel) and 7 dpi (right panel) were plotted and visualized in the form of PCA plot.

To comprehensively evaluate disease outcomes, we performed an integrated analysis that explored the relationship between different viral exposure doses and their effects on various aspects of physiology, pathology, virology, and immunology. The principal components analysis (PCA) plot depicted the normalized values of parameters including body weight loss, viral RNA copies in lung tissue, pathological scores of lung lobes, fold changes of proinflammatory cytokines, and innate antiviral response‐associated genes in lung tissue at 3 and 7 dpi (Figures 5 and S4). As expected, an increase in viral exposure dose was closely correlated with more pronounced body weight loss, severe lung injury, higher levels of viral RNA, upregulation of proinflammatory cytokines, and downregulation of innate antiviral response‐associated genes during both the early and late stages of COVID‐19 progression. This significant finding highlights the importance of controlling viral replication and restoring immune balance in the early stages of COVID‐19 to prevent disease exacerbation in senescent hamsters exposed to high viral doses.

### 2.5 Early intervention with CoVR‐MV is sufficient to save the senescent hamsters from mortality and critical COVID‐19

**FIGURE 6:**
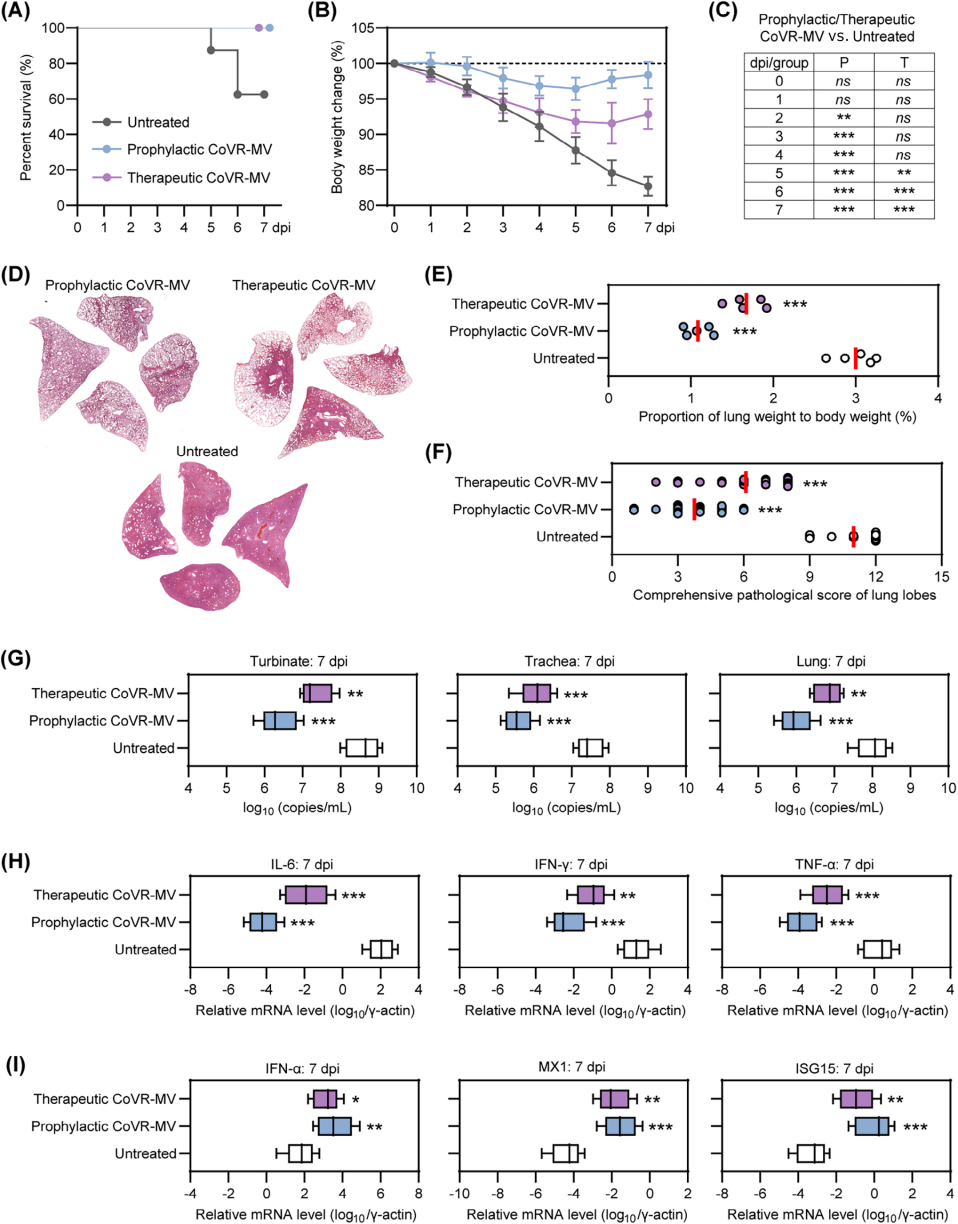
Early administration of CoVR‐MV rescues senescent hamsters from lethal COVID‐19. (A) survival rate (*n* = 10) and (B) changes in body weight (*n* = 5) from 0 to 7 dpi. (C) Statistical analysis was performed to analyze body weight loss data using two‐way ANOVA. (D) Representative H&E staining of the lung lobe sections collected at 7 dpi. Additional H&E staining for the remaining hamsters is shown in Figure S2. (E) Lung weight to body weight ratio was determined at 7 dpi (*n* = 5). (F) Comprehensive pathological scores were assigned to lung sections based on the severity and percentage of injured areas in each lung lobe. Approximately 20 lung lobes were collected from five individual hamsters per group and scored accordingly (Table S2). (G) Viral RNA levels in nasal turbinate, trachea and lung tissues that collected at 7 dpi were quantified by RT‐qPCR (*n* = 5) using primers targeting the SARS‐CoV‐2 ORF1ab gene. Fold changes in the mRNA levels of (H) proinflammatory cytokines including IL‐6, IFN‐γ, and TNF‐α, and (I) IFN‐α signaling‐associated genes in lung tissues that collected at 7 dpi were measured by RT‐qPCR (*n* = 5). These mRNA levels were normalized to the housekeeping gene γ‐actin. (E–I) Significance was determined by one‐way ANOVA for parameters. Two‐sided *p* values < 0.01 were considered statistically significant: *p < 0.01, **p < 0.001, ***p < 0.0001, and ns indicates no significance.

To combat the highly pathogenic coronaviruses, we have recently developed a biomimetic decoy called CoVR‐MV,^24^ which consists of polymerized coronavirus receptors embedded in a biomimetic cell membrane vesicle system. CoVR‐MV has been shown to be effective against SARS‐CoV‐2 infection by directly neutralizing circulating virus particles and exerting spontaneous immunoregulatory effects.^24^ It serves as an ideal option for both prophylactic and therapeutic interventions in the early stages of COVID‐19 progression. In this study, we inoculated senescent hamsters (80‐week‐old males) with 1 × 10^5^ TCID_50_ of SARS‐CoV‐2 Omicron BA.5 and administered a single inhaled dose (1.5 mg/kg) of CoVR‐MV treatment at either −1 or 1 dpi, as previously described. The untreated BA.5‐infected hamsters served as the control group. We then compared the physiological, pathological, virological, and immunological differences among the three groups. Throughout the period from 0 to 7 dpi, three out of eight untreated hamsters died, whereas all hamsters receiving prophylactic or therapeutic CoVR‐MV intervention survived (Figure 6A). Additionally, BA.5‐infected hamsters with prophylactic or therapeutic CoVR‐MV intervention experienced significantly less body weight loss compared with the untreated group, with percentages of 1.8 ± 1.9, 7.1 ± 2.1, and 17.3 ± 1.3% at 7 dpi, respectively (Figure 6B). Therapeutic intervention with CoVR‐MV notably prevented body weight loss from 5 to 7 dpi, although not during the early stages of COVID‐19 progression (Figure 6C). Histological analysis using H&E staining of representative lung tissues collected at 7 dpi revealed that prophylactic CoVR‐MV intervention largely prevented diffuse lung injury, whereas severe lung injury was observed in some lobes of hamsters with therapeutic CoVR‐MV intervention (Figures 6D and S5). Fortunately, both prophylactic and therapeutic CoVR‐MV interventions significantly reduced the lung weight‐to‐body weight ratio, indicating a milder symptom of pulmonary edema (Figure 6E). The average comprehensive pathological scores for BA.5‐infected hamsters receiving prophylactic or therapeutic CoVR‐MV intervention and the untreated group were 3.8 ± 1.5, 6.1 ± 1.9, and 11.0 ± 1.3, respectively (Figure 6F and Table S2). These results demonstrate that early intervention with CoVR‐MV is sufficient to rescue senescent hamsters from critical COVID‐19 after exposure to a high viral dose. Furthermore, hamsters with prophylactic CoVR‐MV intervention exhibited a milder disease index compared with those with therapeutic intervention.

Afterward, we examined the differences in viral RNA in the respiratory organs, as well as proinflammatory cytokines and innate antiviral immune response‐associated genes in the lung tissues of the three groups. Both prophylactic and therapeutic interventions with CoVR‐MV significantly reduced viral RNA levels in respiratory organs, including the turbinates, trachea, and lungs (Figure 6G). The hamsters that received prophylactic intervention with CoVR‐MV had approximately 5–10‐fold lower viral RNA levels in the respiratory tract organs compared with those that received therapeutic intervention with CoVR‐MV (Figure 6G). Moreover, both prophylactic and therapeutic interventions with CoVR‐MV resulted in a significant decrease in the mRNA levels of proinflammatory cytokines, such as IL‐6, IFN‐γ, and TNF‐α (Figure 6H), while inducing a significant increase in the expression of innate antiviral immune response‐associated genes, including IFN‐α, MX1, and ISG15 (Figure 6I). These results suggest a restoration of the dysregulated immune response pattern. Remarkably, hamsters with prophylactic intervention of CoVR‐MV exhibited relatively lower mRNA levels of proinflammatory cytokines and higher mRNA levels of innate antiviral immune response‐associated genes compared with those with therapeutic intervention of CoVR‐MV (Figure 6H,I). In conclusion, these results demonstrate that early intervention with CoVR‐MV is sufficient to rescue senescent hamsters from death and critical COVID‐19.

## 3 DISCUSSION

Vaccines have significantly reduced the severity of SARS‐CoV‐2 infection and its impact on adult health individuals.^29^, ^30^, ^31^ However, global transmission and mutation of the virus pose a challenge to the prevention and treatment of severe COVID‐19 cases in immunocompromised populations, particularly the elderly.^29^ Highly simulated animal models provide valuable tools to improve our understanding of risk factors, key regulators, disease characteristics, and outcomes.^32^, ^33^, ^34^ These models are also useful to investigate the underlying immunopathophysiological mechanisms of SARS‐CoV‐2 infection and to validate potential interventions. The Syrian hamster, known for its susceptibility to the SARS‐CoV and the Middle East respiratory syndrome (MERS‐CoV), serves as a suitable option to mimic the infectivity and pathogenicity of different SARS‐CoV‐2 variants observed in human patients.^35^, ^36^, ^37^, ^38^ Compared with other commonly used animal models such as the human ACE knock‐in mouse, ferret, and nonhuman primate, the hamster model offers advantages such as easy accessibility, low cost, appropriate body size, and high operability in Biosafety Level 3 laboratories. In a previous study, adult hamsters (10 weeks old) successfully resisted exposure to a high dose of the SARS‐CoV‐2 Omicron BA.1 variant, whereas juvenile hamsters (4 weeks old) and older hamsters (60 weeks old) developed critical illness.^39^ This suggests that even the early SARS‐CoV‐2 Omicron variants with lower intrinsic virulence can cause severe cases of COVID‐19 in populations with compromised immune systems. Several parallel studies confirmed the age‐dependent progression of SARS‐CoV‐2 infection and disease outcome in the hamster model. Osterrieder et al.^40^ compared the course of 1 × 10^5^ PFU prototype SARS‐CoV‐2 infection in male and female hamsters in different age groups. Of note, the 9‐week‐old hamsters showed less body weight loss, lower viral load, and rapid lung recovery than the middle‐aged (32–34‐week‐old) hamsters. Ohno et al.^41^ reported that after infection with 1.5 × 10^4^ PFU prototype SARS‐CoV‐2, the middle‐aged hamsters (over 36 weeks old) showed significant body weight loss, prolonged prothrombin time and marked acute kidney injury compared with the 9‐week‐old hamsters, suggesting an age‐dependent risk of extrapulmonary injury.

In this study, we further investigated the effect of viral exposure dose on the disease outcome of SARS‐CoV‐2 Omicron BA.5 infection in senescent hamsters. Our comprehensive analysis revealed a strong correlation between changes in physiology, pathology, virology, and immunology in senescent hamsters and increasing viral exposure dose. Senescent hamsters exposed to a low viral dose exhibited an appropriate innate immune response during the early stages of SARS‐CoV‐2 infection, effectively controlling viral replication and minimizing excessive release of proinflammatory cytokines. This response pattern closely resembled disease characteristics and outcomes observed in asymptomatic and mildly ill COVID‐19 patients. In contrast, senescent hamsters exposed to a high viral dose exhibited typical features of critical COVID‐19, including robust viral replication, inappropriate activation of the IFN signaling pathway, and excessive release of proinflammatory cytokines. These observations are consistent with the results of prototype strain and Omicron BA.1 infection in middle‐aged or elderly hamsters, and further confirm that aging remains a significant risk factor for critical COVID‐19. In contrast to previous studies, the overarching research extends the age of elderly hamsters from 30‐ to 60‐week‐old to 80‐week‐old and evaluates the risk of Omicron BA.5 variant, highlights the critical role of high viral exposure dose as a contributing factor to disease exacerbation in senescent hamsters and attempts to alleviate critical COVID‐19 by performing early intervention of CoVR‐MV.

Over the past 3 years, significant progress has been made in antiviral therapy and immunoregulation as effective approaches to combat SARS‐CoV‐2 infection and COVID‐19 progression.^42^, ^43^, ^44^, ^45^ However, the complex interplay between viral replication and the host immune response poses a challenge to the efficacy of single‐target therapies. During the early stages of COVID‐19, high viral load triggers a transition in the innate immune response from antiviral to proinflammatory, characterized by impaired type I IFN signaling and excessive release of proinflammatory cytokines.^46^, ^47^, ^48^ This immune dysregulation leads to delayed viral clearance, programmed cell death, and extracellular matrix degradation, further exacerbating the unbalanced immune response, resulting in a cytokine storm and diffuse lung injury. In this study, we observed decreased mRNA levels of IFN‐α, MX1, and ISG15 in the lung tissue of senescent hamsters exposed to a high viral dose. These findings suggest insufficient production of type I IFN and blocked activation of downstream functional genes, which play a critical role in early control of robust viral replication. In addition, the increased mRNA levels of IL‐6, IFN‐γ, and TNF‐α in the lung tissues of these hamsters indicated the initiation and progression of a cytokine storm from 3 to 7 dpi.

To address the complex immunopathophysiology changes associated with COVID‐19, we conducted prophylactic and therapeutic interventions using CoVR‐MV during the early stages of the disease. CoVR‐MV is a multifunctional therapeutic platform specifically designed to combat highly pathogenic coronaviruses.^24^ It uses ultrasound to generate ∼200 nm vesicles that carry both the ACE2 receptor protein of SARS‐CoV/SARS‐CoV‐2 and the DPP4 receptor protein of MERS‐CoV on their cell membrane. This innovative design maximizes the spike protein target interface, enabling CoVR‐MV to effectively neutralize a wide range of viruses, including SARS‐CoV, MERS‐CoV, SARS‐CoV‐2, and their circulating variants. In addition, CoVR‐MV has the ability to modulate the imbalanced innate immune response. It does this by promoting the production of endogenous type I IFN through the depression of DHCR7, which inhibits IRF3 in macrophages. In addition, CoVR‐MV effectively suppresses the cytokine storm. In our study, early intervention with CoVR‐MV significantly rescued senescent hamsters from death and critical COVID‐19. It reduced viral mRNA levels in respiratory organs, upregulated IFN‐α signaling, and attenuated excessive release of proinflammatory cytokines. Furthermore, senescent hamsters that received prophylactic intervention with CoVR‐MV at −1 dpi exhibited milder disease outcomes compared with those that received therapeutic intervention at 1 dpi. These findings underscore the importance of implementing prophylactic interventions in the elderly both before and after high‐exposure events. Another notable advantage of the CoVR‐MV is its high editability. By anchoring specific receptor proteins to the carrier cell membrane, the target spectrum of CoVR‐MV can be expanded to include emerging pathogens.

In conclusion, we focused on investigating the physiological, pathological, virological, and immunological changes in the lung tissues of senescent hamsters after exposure to various doses of SARS‐CoV‐2 Omicron BA.5. Through our comprehensive analysis, we found compelling evidence that a high viral exposure dose plays a significant role in the progression of critical COVID‐19, as observed from the disease characteristics and outcomes. Furthermore, we successfully demonstrated that early intervention with CoVR‐MV, which inhibits viral replication and primes innate antiviral immune responses, effectively rescues senescent hamsters from the lethal effects of COVID‐19. These findings contribute significantly to our understanding of the immunopathophysiological mechanisms involved in SARS‐CoV‐2 infection and provide valuable insights into the prevention and treatment of critical COVID‐19 in the elderly population.

Previous clinical and experimental research has provided evidence that sex is a host factor that influences the disease outcome of COVID‐19, with males experiencing more severe symptoms compared with females.^49^, ^50^, ^51^, ^52^ To eliminate the potential impact of sex differences, we used only male senescent hamsters for this study. A subset of critical COVID‐19 patients may progress to multiorgan failure.^53^, ^54^ However, due to time constraints in the Biosafety Level 3 laboratory, we were unable to analyze the physiological, pathological, virological, and immunological changes in the other major organs of the senescent hamsters in this study. However, we collected samples from the liver, heart, spleen, kidney, and brain with the intention of conducting further analysis in future studies.

## 4 MATERIALS AND METHODS

### 4.1 Experimental animal study and sample size

The 80‐week‐old hamsters (LVG strain) used in this study were raised in the specific pathogen free animal feeding facilities. In the study of different inoculation virus doses (group 1: mock; groups 2−5: 1 × 10^2^ TCID_50_, 1 × 10^3^ TCID_50_, 1 × 10^4^ TCID_50_, and 1 × 10^5^ TCID_50_, respectively), eight hamsters were set in each group for observation of survival rate within 7 days. For measurement of the lung pathological changes, viral load, and immunological features, five hamsters in groups 1−5 were euthanized at 7 dpi, respectively. In parallel experiment, seven hamsters were set in groups 2−5 of varied inoculation virus doses, and five hamsters in each group were euthanized at 3 dpi. The extra experimental animals were employed to avoid insufficient sample quantity caused by death during the infection course. In the study of CoVR‐MV intervention, 10 hamsters were set in each group for observation of survival rate within 7 days. All of the surviving hamsters were euthanized at 7 dpi. Because nearly half of the hamsters without therapy died from SARS‐CoV‐2 infection, the data of five hamsters euthanized at 7 dpi in each group were used for the physiology, pathology, virology, and immunology analysis.

### 4.2 System evaluation of disease outcome

Detailed information of biosafety operations, virus stock, virus inoculation, symptom observation, sample collection, detection of viral RNA, measurement of cytokine mRNA, histopathological studies, and statistical analysis were shown in the Supporting Information File.

## AUTHOR CONTRIBUTIONS

*Xuan Liu, Ming Zhou, Mujing Fang and Ying Xie*: contributed equally to this work. *Huachen Zhu and Peiwen Chen*: provided virus stock. *Ming Zhou, Rirong Chen, Jianghui Ye, and Kun Wu*: performed animal studies and sample detection. *Xuan Liu*: provided the CoVR‐MV. *Mujing Fang, Ying Xie, and Che Liu* participated in project design and data analysis. *Xuan Liu and Lunzhi Yuan*: wrote the manuscript. *Lunzhi Yuan, Tong Cheng, Hui Zhao, Yi Guan, and Ningshao Xia*: supervised this study. All authors have read and approved the final manuscript.

## CONFLICT OF INTEREST STATEMENT

The authors declare no conflict of interests.

## ETHICS STATEMENT

In this study, the virus and animal studies were approved by the ethics committee of the Guangdong‐Hong Kong Joint Laboratory of Emerging Infectious Diseases/Joint Laboratory for International Collaboration in Virology and Emerging Infectious Diseases (Key Laboratory of Ministry of Education), Joint Institute of Virology (Shantou University/The University of Hong Kong) (Approval number: SUMC2022‐053).

## Supporting information

Supporting Information

## Data Availability

The data that support the findings of this study are available from the corresponding author upon reasonable request.
