# Supplementary material for: Decisive reversal of lethal coronavirus disease 2019 in senescent hamster by synchronic antiviral and immunoregulatory intervention
Source: MedComm (2020). 2024 Jul 19;5(8):e642. doi: 10.1002/mco2.642 (PMC11258460; doi:10.1002/mco2.642)
Supplement: Supplementary file 1 — Supporting Information [file MCO2-5-e642-s001.docx]

**Supporting Data**

**Decisive reversal of lethal COVID-19 in senescent hamster by synchronic antiviral and immunoregulatory intervention**

Xuan Liu, Ming Zhou, Mujing Fang, Ying Xie, Peiwen Chen, Rirong Chen, Kun Wu, Jianghui Ye, Che Liu, Huachen Zhu, Tong Cheng, Lunzhi Yuan, Hui Zhao, Yi Guan, Ningshao Xia

**Detailed materials and methods**

**Biosafety Operations**

All experiments with infectious SARS-CoV-2 were performed in the biosafety level 3 (BSL-3) and animal biosafety level 3 (ABSL-3) facilities. Our staff wore powered air-purifying respirators that filtered the air, and disposable coveralls when they cultured the virus and handled animals that were in isolators. The researchers were disinfected before they left the room and then showered on exiting the facility. All facilities, procedures, training records, safety drills, and inventory records were subject to periodic inspections and ongoing oversight by the institutional biosafety officers who consult frequently with the facility managers.

**Virus Stock**

The SARS-CoV-2 Omicron BA.5 variant AP-101 (share an identical sequence with EPI_ISL_12920651) was passaged on Vero cells (#CCL-81, ATCC). Viral stocks were prepared in Vero cells with DMEM containing 2% FBS, 5ug/mL TPCK-trypsin, 1% Penicillin-Streptomycin and 30mmol/L MgCl_2_. Viruses were harvested and stored in ultra-low temperature freezer. The titers were determined by means of plaque assay in Vero cells.

**Virus Inoculation, Symptom Observation and Sample Collection**

The 80-week-old male hamsters were anesthetized by isoflurane (#R510-22, RWD Life Science) and nasally inoculated with indicated doses of SARS-CoV-2 diluted in 200μL of PBS (#10010031, GIBCO). In the next seven days, body weight of these hamsters was measured by an electronic balance. Meanwhile, the hamsters were daily observed for illness symptoms of weakness and abdominal respiration during the weighting. Each symptom was scored based on the severity of none (0), moderate (1), mild (2), severe (3) and very severe (4), respectively. For the symptom of weakness, 0 indicates health, 1 indicates inactive, 2/3 indicates slight and significant trembling, 4 indicates partial or complete loss of mobility. For the symptom of abdominal respiration, 0 indicates normal respiration, 1/2 indicates mild and significant shortness of breath, 3/4 indicates mild and significant degrees of Abdominal breath. Hamsters were euthanized at the indicated time point for detection of viral load in respiratory tract organs and pathological examination in lung lobes.

**Detection of Viral RNA**

For the solid organ samples, we collect 1mg turbinate, 0.1 mg trachea and 0.1 mg lung in 1mL PBS for homogenate and detection of viral RNA and viral titer. Viral RNA was extracted by using a QIAamp Viral RNA Mini kit (#52906, Qiagen) according to the manufacturer's instructions. The RT-qPCR was conducted by using the SLAN-96S Real-Time System (Hongshi, Shanghai, China) with a SARS-CoV-2 RT-qPCR Kit from Wantai (Beijing, China). Relative Viral RNA of SARS-CoV-2 ORF1ab gene was determined using primer pairs and probes provided in the kit. Viral RNA copies were expressed on a log_10_ scale after normalized to the standard curve obtained by using ten-fold dilutions of a SARS-CoV-2 stock.

**Measurement of Cytokine mRNA**

The lung tissues were cleaved into small pieces and soaked in RNAlater (#AM7021, Invitrogen). Total RNAs in lysed lung tissues were extracted with RNeasy Mini kit (#74106, Qiagen) and reverse-transcribed to cDNA with Fast-King Strand cDNA Synthesis Kit (#FP313, TIANGEN, Beijing) Diluted cDNAs (1:10) were quantified using SYBR Green I-based RT-qPCR using the LightCycler® 480 instrument (Roche) per manufacturer’s instructions. Threshold cycle (Ct) of each gene was normalized to the internal reference gene (hamster γ-actin) and comparative Ct (2-ΔΔCt) method was utilized to calculate changes in chemokine and cytokine gene expression profile. The gene-specific primers (5’ to 3’) used for RT-qPCR were listed in Supplementary Table S3.

**Histopathological Studies**

For pathological analysis, lung tissues were fixed in formalin for more than 72 hours, dehydrated and then embedded in paraffin wax. The wax block of lung tissues was cut into 4μm sections for pathological staining and analysis. H&E staining was employed for analysis of general lung pathogenic lesions including pulmonary edema, consolidation, and inflammation. The standards for pathological score of lung tissues in this study are derived from our previous study in hamster model. Comprehensive pathological scoring of lung sections was performed according to the degree of lung lesions including alveolar septum hyperplasia, consolidation and impairment of alveolar structure, fluid exudation, mucus suppository, thrombus, inflammation recruitment and infiltration of immune cells in each individual lung lobe. For each hamster, three or four lung lobes were employed for evaluation of comprehensive pathological score. In brief, H&E staining result of each lung lobe was analyzed for its severity of pathological change. The pathological score included: a) Alveolar septum thickening and consolidation; b) Hemorrhage, exudation, pulmonary edema and mucous; c) Recruitment and infiltration of inflammatory immune cells. For each issue, scores were related to the severity: 0 indicated no pathological change was observed, 1 indicated moderate pathological change, 2 indicated mild pathological change, 3 indicated severe pathological change and 4 indicated very severe pathological change. In conclusion, scores of such three issues were added as the comprehensive pathological score of a lung lobe, and the average comprehensive pathological score of the lobes indicated the severity of lung pathology in an evaluated hamster. The pathological reagents include Hematoxylin (#CTS-1096) and Eosin (#CTS-4094) were purchased from Maxim Biotechnology (Fuzhou, China). The images of whole lung lobes were screened by a high-throughput screening microscope system (EVOS M7000, Invitrogen of Thermo-Fisher Scientific).

**Statistical Analysis**

Student’s unpaired two-tailed t-test, one-way ANOVA and were performed using GraphPad Prism 8.0 (GraphPad Software). Data are presented as the means ± SD. Two-sided p-values <0.01 were considered significant: *P <0.01, **P <0.001, ***P <0.0001, *ns* indicates no significance.


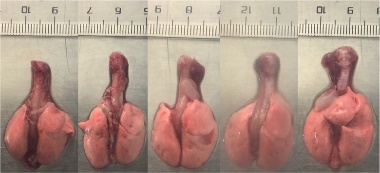


**Figure S1.** Gross images of lung lobes collected from mock hamsters (group 1) at 7 dpi, related to Figure 2.


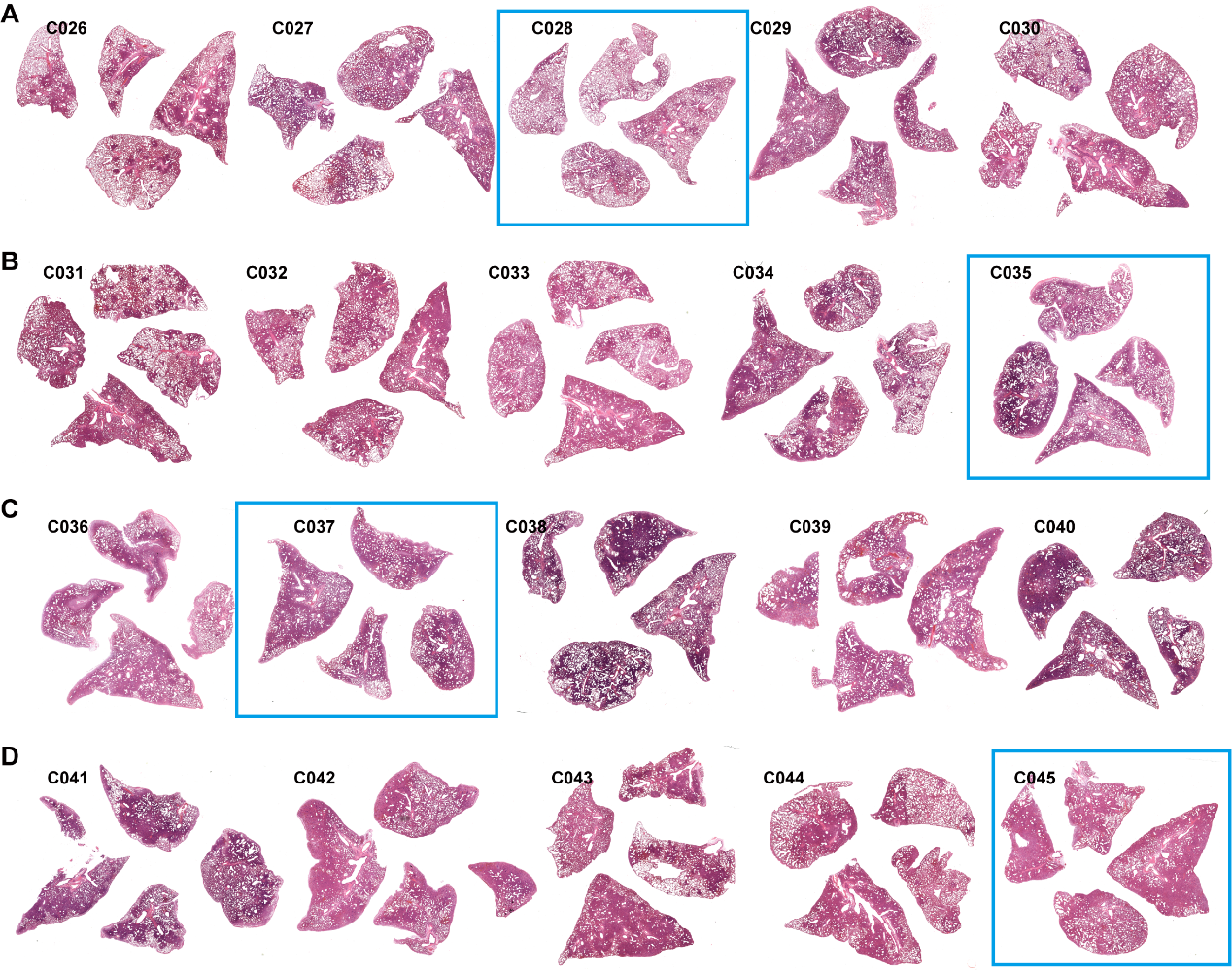


**Figure S2.** H&E staining of lung lobes collected from BA.5-infected hamsters that sacrificed at 5 dpi, related to Figure 2. For each hamster, three or four lung lobes were fixed in formalin for pathological analysis. Overall screening images for the H&E staining lung lobe sections from the hamsters in group **(A)** 2, **(B)** 3, **(C)** 4 and **(D)** 5 were displayed, respectively. The representative lung lobes in blue frames were displayed in Figure 2C.


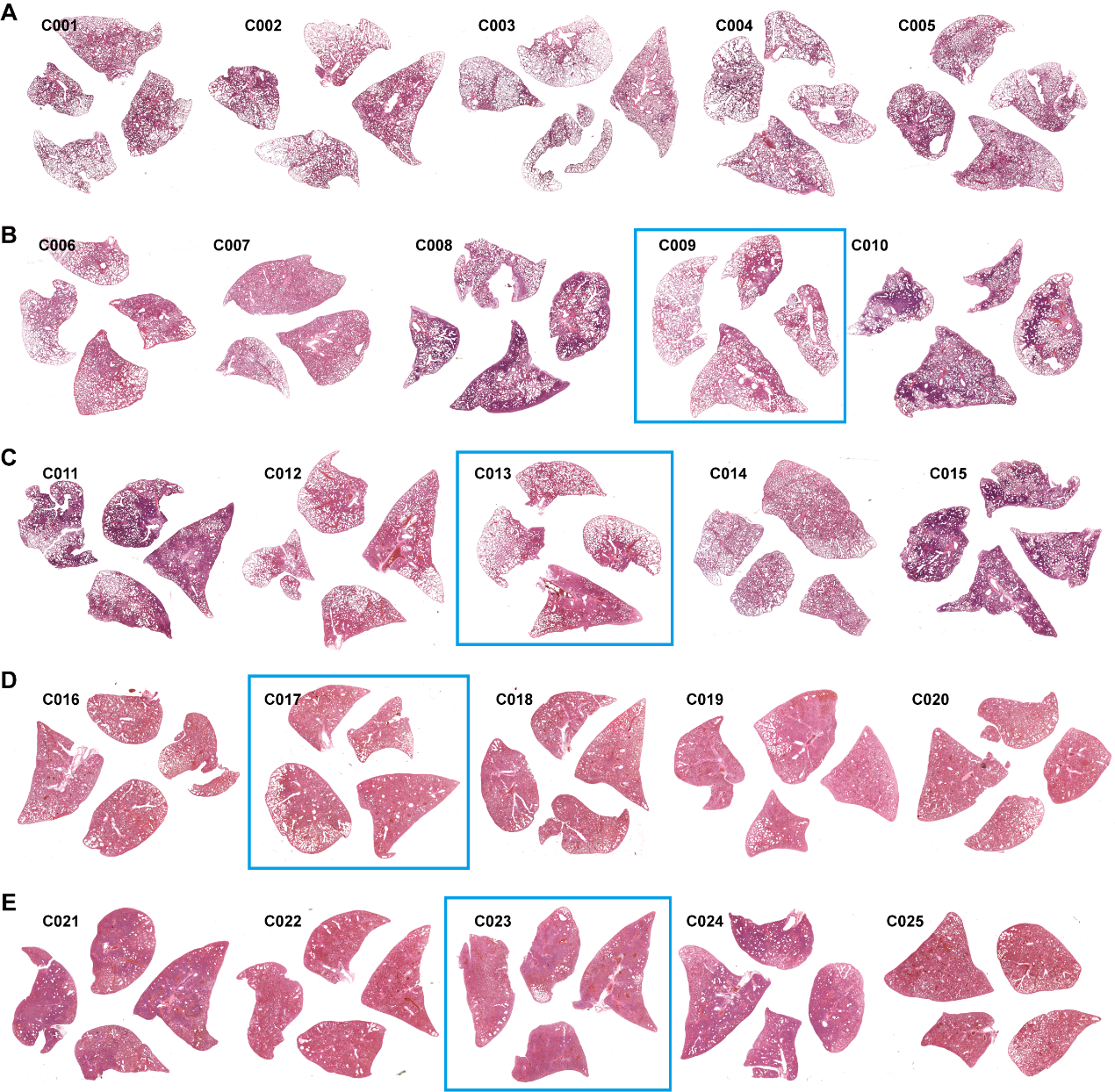


**Figure S3.** H&E staining of lung lobes collected from the mock hamsters and BA.5-infected hamsters that sacrificed at 7 dpi, related to Figure 2. For each hamster, three or four lung lobes were fixed in formalin for pathological analysis. Overall screening images for the H&E staining lung lobe sections from the hamsters in group **(A)** 1, **(B)** 2, **(C)** 3, **(D)** 4 and **(E)** 5 were displayed, respectively. The representative lung lobes in blue frames were displayed in Figure 2C.


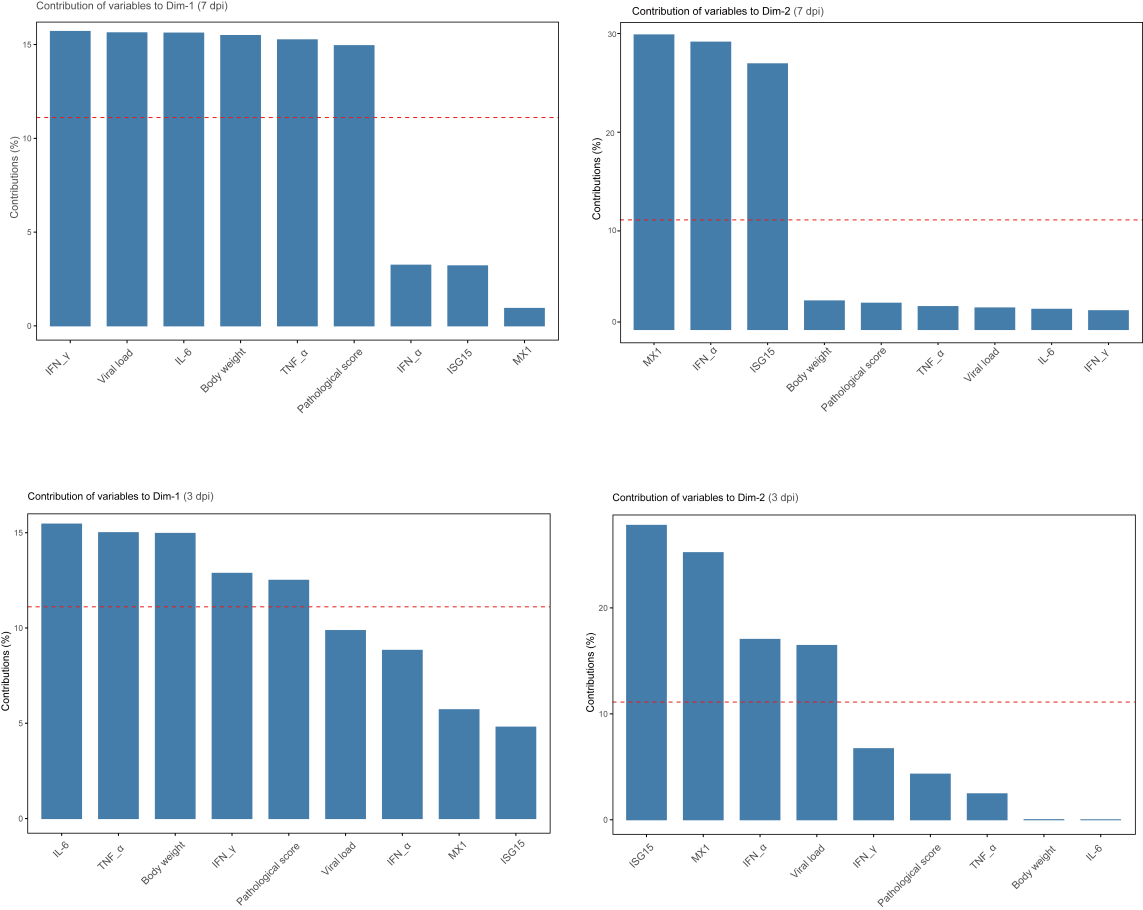


**Figure S4.** The contribution of the nine variables to the first/second principal component, with higher values indicating a stronger explanation of the variable for the principal component.


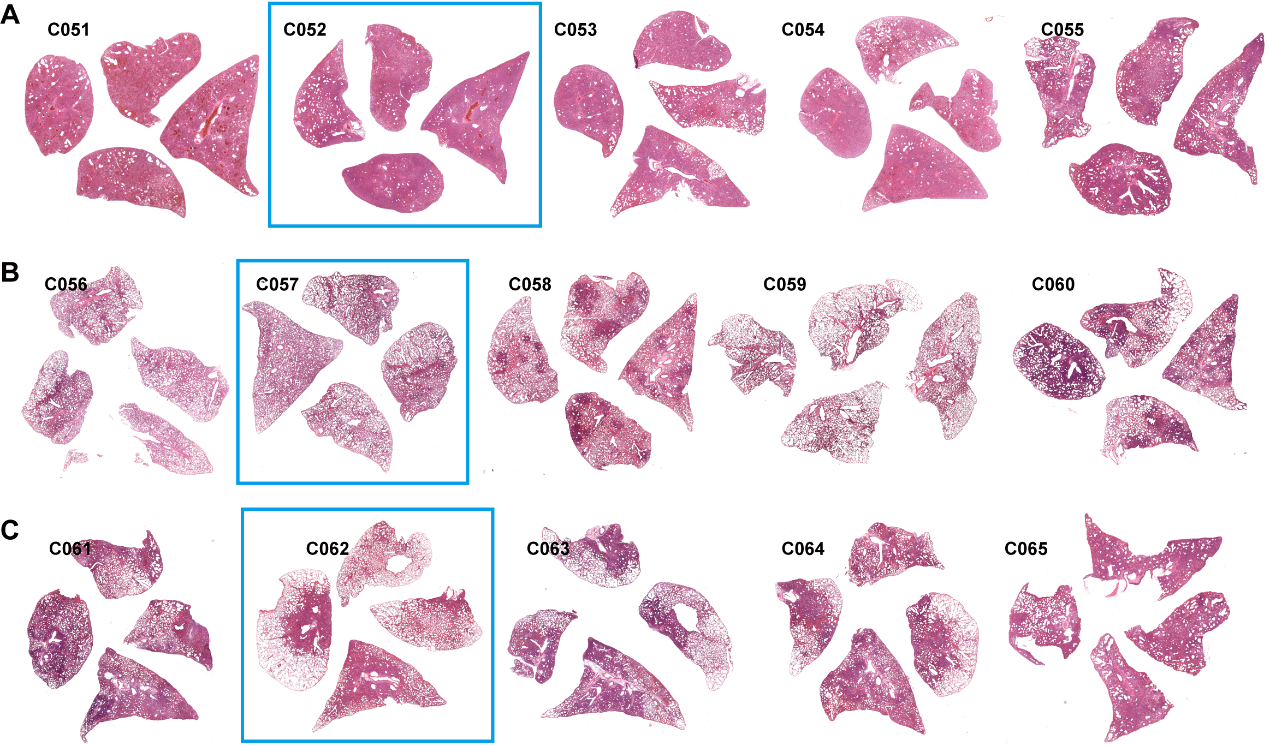


**Figure S5.** H&E staining of lung lobes collected from the BA.5-infected hamsters with or without CoVR-MV intervention, related to Figure 6. All of the hamsters were sacrificed at 7 dpi. For each hamster, three or four lung lobes were fixed in formalin for pathological analysis. Overall screening images for the H&E staining lung lobe sections from the **(A)** control hamsters (untreated) and those with early intervention of CoVR-MV at **(B)** -1 or **(C)** 1 dpi were displayed. The representative lung lobes in blue frames were displayed in Figure 6D.

**Table S1. Comprehensive pathological scores for the lung lobes of BA.5-infected hamsters in groups 1 to 5 that sacrificed at 3 and 7 dpi, related to Figure 2.**

| **Group** | **No.** | **Pathological lesions** | | | **Comprehensive pathological score** |  |
| --- | --- | --- | --- | --- | --- | --- |
|  |  |  |  |  |  |  |
|  |  | **Alveolar septum hyperplasia and consolidation** | **Pulmonary edema, hemorrhage and mucus suppository** | **Recruitment and infiltration of inflammatory cells** |  |  |
|  |  |  |  |  |  |  |
| **1×10E2 TCID50 (3 dpi)** | **#C026** | 2+1+1+1 | 2+2+2+2 | 2+1+1+1 | 6+4+4+4 |  |
|  | **#C027** | 2+1+1+1 | 2+2+2+1 | 2+2+2+1 | 6+5+5+3 |  |
|  | **#C028** | 1+1+0+0 | 1+1+1+1 | 1+1+0+0 | 3+3+1+1 |  |
|  | **#C029** | 2+2+2+2 | 2+2+2+2 | 3+3+2+2 | 7+7+6+6 |  |
|  | **#C030** | 3+2+1+1 | 2+2+2+1 | 3+2+2+1 | 8+6+5+3 |  |
| **1×10E3 TCID50 (3 dpi)** | **#C031** | 3+2+1+1 | 3+2+1+1 | 3+2+1+1 | 9+6+3+3 |  |
|  | **#C032** | 3+2+1+1 | 3+2+2+2 | 3+2+2+2 | 9+6+5+5 |  |
|  | **#C033** | 3+2+1+1 | 3+2+2+2 | 3+2+1+1 | 9+6+4+4 |  |
|  | **#C034** | 3+3+1+1 | 3+3+2+2 | 3+3+2+1 | 9+9+5+4 |  |
|  | **#C035** | 2+2+1+1 | 3+2+2+2 | 3+2+2+2 | 8+6+5+5 |  |
| **1×10E4 TCID50 (3 dpi)** | **#C036** | 3+3+3+1 | 3+3+2+1 | 3+3+3+0 | 9+9+8+2 |  |
|  | **#C037** | 3+2+2+2 | 3+2+2+2 | 3+3+3+3 | 9+7+7+7 |  |
|  | **#C038** | 3+1+1+1 | 3+2+2+2 | 4+2+2+1 | 10+5+5+4 |  |
|  | **#C039** | 3+2+2+2 | 3+3+2+2 | 3+3+3+3 | 9+8+7+7 |  |
|  | **#C040** | 3+1+1+1 | 3+3+2+2 | 4+2+2+2 | 10+6+5+5 |  |
| **1×10E5 TCID50 (3 dpi)** | **#C041** | 3+3+2+2 | 2+2+2+2 | 3+3+2+2 | 8+8+6+6 |  |
|  | **#C042** | 3+3+2+2 | 2+2+2+2 | 4+3+3+3 | 9+8+7+7 |  |
|  | **#C043** | 3+2+2+2 | 3+2+2+2 | 4+3+2+2 | 10+7+6+6 |  |
|  | **#C044** | 3+2+1+1 | 3+3+1+1 | 3+2+2+2 | 9+7+4+4 |  |
|  | **#C045** | 4+3+2+2 | 3+3+2+2 | 4+3+2+2 | 11+9+6+6 |  |
| **1×10E2 TCID50 (7 dpi)** | **#C006** | 3+2+1+1 | 2+2+1+1 | 3+2+1+0 | 8+6+3+2 |  |
|  | **#C007** | 3+3+1 | 3+3+1 | 2+2+0 | 8+8+2 |  |
|  | **#C008** | 1+1+1+0 | 2+2+2+1 | 2+2+1+0 | 5+5+4+1 |  |
|  | **#C009** | 1+1+1+0 | 2+1+1+1 | 1+1+0+0 | 4+3+1+1 |  |
|  | **#C010** | 1+1+1+1 | 2+2+2+1 | 2+2+2+1 | 5+5+5+3 |  |
| **1×10E3 TCID50 (7 dpi)** | **#C011** | 3+2+1+1 | 2+2+1+1 | 3+2+2+1 | 9+6+4+3 |  |
|  | **#C012** | 2+1+1+1 | 3+2+2+2 | 3+2+1+1 | 9+5+4+4 |  |
|  | **#C013** | 3+1+1+1 | 3+1+1+1 | 4+1+1+1 | 10+3+3+3 |  |
|  | **#C014** | 2+1+1+1 | 3+3+3+1 | 0+0+0+0 | 5+4+4+2 |  |
|  | **#C015** | 2+1+1+1 | 2+2+1+1 | 3+2+1+1 | 7+5+3+3 |  |
| **1×10E4 TCID50 (7 dpi)** | **#C016** | 4+4+4+3 | 4+4+4+4 | 1+1+1+1 | 9+9+9+8 |  |
|  | **#C017** | 4+4+4+4 | 4+4+3+3 | 1+1+1+1 | 9+9+8+8 |  |
|  | **#C018** | 4+4+4+3 | 4+4+4+4 | 2+1+1+1 | 10+9+9+8 |  |
|  | **#C019** | 4+4+4+3 | 4+4+4+4 | 2+2+2+1 | 10+10+10+8 |  |
|  | **#C020** | 4+4+3+3 | 4+4+2+2 | 2+2+1+1 | 10+10+6+6 |  |
| **1×10E5 TCID50 (7 dpi)** | **#C021** | 4+4+4+3 | 4+4+4+3 | 4+4+4+2 | 12+12+12+8 |  |
|  | **#C022** | 4+4+4+4 | 4+4+4+4 | 2+2+2+2 | 10+10+10+10 |  |
|  | **#C023** | 4+4+4+4 | 4+4+4+3 | 4+4+4+4 | 12+12+12+11 |  |
|  | **#C024** | 4+4+4+4 | 3+3+3+3 | 4+4+4+4 | 11+11+11+11 |  |
|  | **#C025** | 4+4+2+2 | 4+4+4+2 | 2+2+2+1 | 10+10+8+5 |  |
| **Mock (7dpi)** | **#C001** | 1+1+1+0 | 2+2+2+1 | 1+1+1+0 | 4+4+4+1 |  |
|  | **#C002** | 2+1+1+1 | 2+2+1+1 | 2+1+0+0 | 6+4+2+2 |  |
|  | **#C003** | 1+0+0+0 | 1+1+1+1 | 0+0+0+0 | 2+1+1+1 |  |
|  | **#C004** | 1+1+0+0 | 2+2+1+1 | 1+0+0+0 | 4+3+1+1 |  |
|  | **#C005** | 1+1+1+1 | 2+2+1+1 | 2+2+0+0 | 5+5+2+2 |  |

**Table S2. Comprehensive pathological scores for the lung lobes of BA.5-infected hamsters with or without early intervention of CoVR-MV that sacrificed at 7 dpi, related to Figure 6.**

| **Group** | **No.** | **Pathological lesions** | | | **Comprehensive pathological score** |  |
| --- | --- | --- | --- | --- | --- | --- |
|  |  |  |  |  |  |  |
|  |  | **Alveolar septum hyperplasia and consolidation** | **Pulmonary edema, hemorrhage and mucus suppository** | **Recruitment and infiltration of inflammatory cells** |  |  |
|  |  |  |  |  |  |  |
| **Untreated (7 dpi)** | **#C051** | 4+4+4+3 | 4+4+4+4 | 4+4+4+4 | 12+12+12+11 |  |
|  | **#C052** | 4+4+4+3 | 4+4+4+3 | 4+4+4+4 | 12+12+12+10 |  |
|  | **#C053** | 4+4+3+3 | 4+4+4+3 | 4+4+4+3 | 12+12+11+9 |  |
|  | **#C054** | 4+4+4+3 | 4+4+4+3 | 4+4+4+3 | 12+12+12+9 |  |
|  | **#C055** | 4+3+3+3 | 3+3+3+3 | 4+3+3+3 | 11+9+9+9 |  |
| **Prophylactic CoVR-MV (7 dpi)** | **#C056** | 1+1+1+1 | 2+2+1+1 | 1+1+1+0 | 4+4+3+2 |  |
|  | **#C057** | 1+1+1+0 | 2+2+1+1 | 1+1+1+0 | 4+4+3+1 |  |
|  | **#C058** | 2+1+1+1 | 2+2+2+1 | 2+2+2+1 | 6+5+5+3 |  |
|  | **#C059** | 1+1+1+0 | 2+2+2+1 | 0+0+0+0 | 3+3+3+1 |  |
|  | **#C060** | 2+2+2+2 | 1+1+1+1 | 3+2+2+2 | 6+5+5+5 |  |
| **Therapeutic CoVR-MV (7 dpi)** | **#C061** | 3+2+2+1 | 2+2+2+2 | 3+3+2+2 | 8+7+6+5 |  |
|  | **#C062** | 3+2+1+1 | 3+1+1+1 | 2+2+1+0 | 8+5+3+2 |  |
|  | **#C063** | 2+2+2+1 | 2+2+2+0 | 3+3+2+2 | 7+7+6+3 |  |
|  | **#C064** | 2+2+2+2 | 3+2+2+2 | 3+3+2+2 | 8+7+6+6 |  |
|  | **#C065** | 3+3+3+1 | 2+2+2+1 | 3+3+3+2 | 8+8+8+4 |  |

**Table S3. The gene-specific primers (5’ to 3’) used for RT-PCR for hamster specific genes, related to Figure 4 and 6.**

| **Genes** | **Forward** | **Reverse** |
| --- | --- | --- |
| **Hamster IFN-γ** | TGTTGCTCTGCCTCACTCAGG | AAGACGAGGTCCCCTCCATTC |
| **Hamster IL-6** | AGACAAAGCCAGAGTCATT | TCGGTATGCTAAGGCACAG |
| **Hamster TNF-α** | TGAGCCATCGTGCCAATG | AGCCCGTCTGCTGGTATCAC |
| **Hamster IFN-α** | CTGGTGGCTGTGAGGAAATA | AGCAAGTTGGCTGAGGAAGA |
| **Hamster ISG15** | AAAGCCTACAGCCATGACCT | TTAGTCAGGGGCACCAGGAA |
| **Hamster MX1** | GCGCTTCCAGACTCTTCTGA | CCTAAGATACATGCGATGGCG |
| **Hamster γ-actin** | ACAGAGAGAAGATGACGCAGATAATG | GCCTGA ATGGCCACGTACA |
